# Increased Levels of hsa-miR-199a-3p and hsa-miR-382-5p in Maternal and Neonatal Blood Plasma in the Case of Placenta Accreta Spectrum

**DOI:** 10.3390/ijms252413309

**Published:** 2024-12-11

**Authors:** Angelika V. Timofeeva, Ivan S. Fedorov, Anastasia D. Nikonets, Alla M. Tarasova, Ekaterina N. Balashova, Dmitry N. Degtyarev, Gennady T. Sukhikh

**Affiliations:** National Medical Research Center for Obstetrics, Gynecology and Perinatology Named After Academician Kulakov V.I., 117997 Moscow, Russia

**Keywords:** placenta accreta spectrum, RDS, antenatal corticosteroid therapy, miRNA, deep sequencing, PCR, neonatal complication, blood plasma

## Abstract

Despite the increasing number of placenta accreta spectrum (PAS) cases in recent years, its impact on neonatal outcomes and respiratory morbidity, as well as the underlying pathogenetic mechanism, has not yet been extensively studied. Moreover, no study has yet demonstrated the effectiveness of antenatal corticosteroid therapy (CT) for the prevention of respiratory distress syndrome (RDS) in newborns of mothers with PAS at the molecular level. In this regard, microRNA (miRNA) profiling by small RNA deep sequencing and quantitative real-time PCR was performed on 160 blood plasma samples from preterm infants (gestational age: 33–36 weeks) and their mothers who had been diagnosed with or without PAS depending on the timing of the antenatal RDS prophylaxis. A significant increase in hsa-miR-199a-3p and hsa-miR-382-5p levels was observed in the blood plasma of the newborns from mothers with PAS compared to the control group. A clear trend toward the normalization of hsa-miR-199a-3p and hsa-miR-382-5p levels in the neonatal blood plasma of the PAS groups was observed when CT was administered within 14 days before delivery, but not beyond 14 days. Direct correlations were found among the hsa-miR-382-5p level in neonatal blood plasma and the hsa-miR-199a-3p level in the same sample (r = 0.49; *p* < 0.001), the oxygen requirements in the NICU (r = 0.41; *p* = 0.001), the duration of the NICU stay (r = 0.31; *p* = 0.019), and the severity of the newborn’s condition based on the NEOMOD scale (r = 0.36; *p* = 0.005). Logistic regression models based on the maternal plasma levels of hsa-miR-199a-3p and hsa-miR-382-5p predicted the need for cardiotonic therapy, invasive mechanical ventilation, or high-frequency oscillatory ventilation in newborns during the early neonatal period, with a sensitivity of 95–100%. According to the literary data, these miRNAs regulate fetal organogenesis via IGF-1, the formation of proper lung tissue architecture, surfactant synthesis in alveolar cells, and vascular tone.

## 1. Introduction

Abnormal placental implantation occurs when trophoblasts invade the superficial uterine endometrium (placenta accreta), the myometrium (placenta increta), or beyond the uterine serosa (placenta percreta). Collectively, these conditions are referred to as placenta accreta spectrum (PAS). The primary cause of PAS is thought to be defective decidualization at the implantation site, leading to the absence of both the decidua basalis and Nitabuch’s layer. This results in the direct attachment of chorionic villi to the myometrium [1,2]. The incidence of PAS is estimated to be as high as 1.1% of all births [3], and this rate is rising globally due to an increase in cesarean deliveries and other uterine surgeries, such as surgical uterine evacuations, myomectomies, and infertility treatments [4,5]. Among the types of PAS, placenta accreta is the most common. In a pooled analysis of hysterectomy specimens with confirmed abnormal placentation, the distribution was as follows: placenta accreta (79%), placenta increta (14%), and placenta percreta (7%) [6].

Several clinical studies have shown that PAS is associated with an increased incidence of respiratory distress syndrome (RDS) and a greater need for neonatal respiratory support, including continuous positive airway pressure [7,8]. RDS occurs due to surfactant deficiency and immature lung development. Although it is well known that preterm infants (those born before 37 weeks of gestation) are at higher risk for RDS, especially those born before 32 weeks [9,10], and that the risk decreases with increasing gestational age as organ systems mature [11,12], earlier analysis at the V.I. Kulakov National Medical Research Center of Obstetrics, Gynecology, and Perinatology revealed a more severe course of the early neonatal period and a higher incidence of RDS in preterm infants born to mothers with PAS compared to those born to mothers without PAS [13]. Despite the increasing number of PAS cases in recent years, its impact on neonatal outcomes and respiratory morbidity has not yet been extensively studied in large multicenter clinical trials. Therefore, it is crucial to understand the underlying mechanisms of neonatal complications in the context of PAS.

Corticosteroids have become the standard of care for women at risk of preterm birth before 32 to 34 weeks of gestation [14] and within the 34–37-week period [15] in many countries. In the fetal lungs, corticosteroids stimulate the production of proteins, promote the biosynthesis of phospholipids, and increase the production of surfactants [16]. Despite the widespread use of antenatal corticosteroids to prevent RDS in preterm infants, there is still no consensus on the optimal corticosteroid type, dosage, frequency, timing, or administration route [17].

The effectiveness of antenatal corticosteroids for preventing RDS in late preterm infants (34/0–36/6 weeks) born to mothers with placenta accreta was specifically assessed at the Kulakov National Medical Research Center of Obstetrics, Gynecology, and Perinatology [18]. The study found that when corticosteroids were administered no later than seven days before delivery, there was a reduction in the severity of respiratory disorders and a decrease in the need for invasive respiratory therapy, including high-frequency oscillatory ventilation (HFOVL).

Despite these findings, no study has yet demonstrated the effectiveness of antenatal corticosteroid therapy in the newborns of mothers with PAS at the molecular level. In the earlier research, we identified microRNA (miRNA) markers of PAS in the blood of women during the first trimester of pregnancy [19] and near the time of delivery [20]. MiRNAs are small non-coding RNAs that are 18–25 nucleotides long that regulate the expression of up to 60% of all protein-coding genes post-transcriptionally [21,22] by destabilizing mRNA or by suppressing translation [23]. MiRNAs perform their function by the complementary binding of the seed region (2–8 nt from the 5′ end of the miRNA) and, sometimes, the supplementary region (13–16 nt from the 5′ end of the miRNA) to specific recognition sites located in the 3′-untranslated regions of the mRNA of target genes as a part of the RNA-induced silencing complex [24,25]. miRNAs have unique multifunctionality, where one miRNA has a wide range of mRNA targets and can therefore control several cellular signaling cascades at once [24]. On the other hand, one target mRNA can bind several different miRNAs at once, which leads to the formation of a complex regulatory network that controls physiological processes in the cell both in normal conditions and in pathology. MiRNAs control all fundamental cellular processes such as proliferation, differentiation, apoptosis, migration, and adhesion [26]. In reproductive biology, miRNAs are involved in processes such as spermatogenesis, folliculogenesis, endometrial functions, embryogenesis, maternal recognition of pregnancy, embryo implantation, and placental development [27,28,29,30]. Aberrant miRNA expression has been linked to numerous pathological conditions, including pregnancy complications [31,32,33]. Their ability to be secreted into biological fluids, combined with their measurability, sensitivity, and stability (average half-life of 119 h), makes them promising markers for identifying pathological conditions [34,35].

In this study, we aimed to investigate whether there are changes in the blood plasma miRNA levels of premature infants born to mothers with PAS compared to infants of similar gestational age born to mothers without PAS. Additionally, we explored whether these changes are associated with the morphological type of PAS, the severity of respiratory and cardiovascular disorders in the newborn, and the timing of antenatal RDS prophylaxis.

## 2. Results

### 2.1. Deep Sequencing of Neonatal Blood Plasma miRNA

In the initial phase of the study, a deep sequencing method was employed to analyze the miRNA profiles in the blood plasma of day-old newborns, aiming to identify differences based on the presence or absence of PAS.

**Figure 1:**
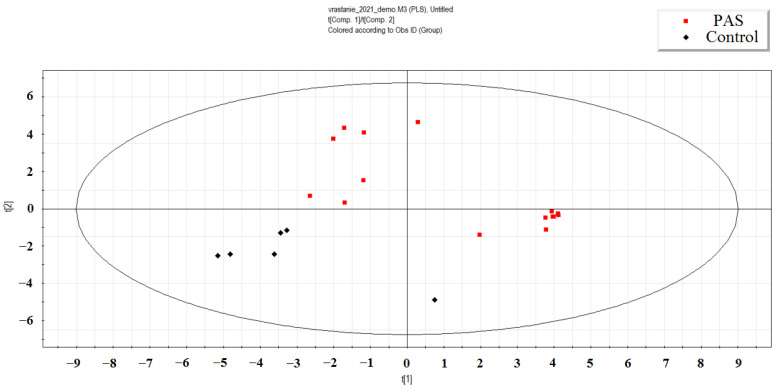
PLS-A analysis of deep sequencing data of miRNA in the peripheral blood plasma of day-old newborns from mothers with PAS and without PAS (control).

Using the partial least squares regression (PLS-A) method, a distinct cluster of neonatal plasma samples from the mothers with PAS and with different timings of antenatal CT was observed, separate from the cluster of samples from the mothers without PAS and without antenatal CT (Figure 1). The most significant contribution to this separation came from the read counts of 42 miRNAs, each with a Variable Importance in Projection (VIP) score greater than 1. The separation was primarily driven by the following 42 miRNAs: hsa-miR-152-3p, miR-339-3p, miR-675-3p, miR-34c-5p, miR-199a-5p, miR-22-3p, miR-625-5p, miR-625-3p, miR-6511a, miR-101-3p, miR-324-3p, let-7d-5p, miR-339-5p, miR-199a-3p, miR-199b-3p, miR-382-5p, miR-1908-5p, miR-382-3p, miR-30c-5p, miR-485-5p, let-7g-5p, let-7f-5p, miR-493-5p, let-7d-3p, miR-136-3p, miR-330-3p, miR-98-5p, miR-335-3p, miR-127-3p, miR-432-5p, miR-205-5p, miR-1180-3p, miR-1306-3p, miR-326, miR-379-5p, miR-3131, miR-26b-5p, miR-320d, miR-421, miR-3180-3p, and miR-6842-3p, miR-195-3p.

Then, we aimed to identify miRNA markers of the pathogenetic effect of PAS on neonatal complications, without the effect of neonatal RDS prophylaxis, among the 42 miRNAs mentioned above. To enhance the significance of these markers, an alternative method of bioinformatics analysis was used (Wilcoxon–Mann–Whitney U test), where a group of newborns of the mothers without PAS and without CT was compared with the PAS group with CT more than 14 days before delivery, excluding the PAS group with CT 2–7 days before delivery, since it was revealed that antenatal corticosteroid therapy to reduce the incidence of RDS is effective for up to seven days after treatment [18,36]. Significant differences were observed between the groups compared in the levels of 38 miRNAs (see Table 1).

Notably, this list includes seven miRNAs (hsa-miR-199a-3p, hsa-miR-199b-3p, hsa-miR-382-5p, hsa-let-7g-5p, hsa-let-7f-5p, hsa-let-7d-3p, hsa-miR-26b-5p) that contributed to the separation of two clusters based on the presence or absence of PAS, as determined by PLS-A analysis (Figure 1). Two miRNAs, hsa-miR-199a-3p and hsa-miR-382-5p, were selected for validation of the quantitative real-time RT-PCR sequencing data across all 160 maternal and neonatal plasma samples collected.

### 2.2. Validation of miRNAs Sequencing Data by Quantitative Real-Time PCR

Before performing quantitative real-time PCR to validate the sequencing data, the clinical parameters of the newborns from mothers that were categorized into five groups (Table 2) by the presence or absence of PAS and the timing of antenatal corticosteroid therapy (CT) were analyzed. All groups with PAS were compared to those without PAS and without CT (Table 2). A significant increase in the weight of a day-old newborn was found in all the groups of newborns from mothers with PAS, except one with CT more than 14 days before delivery. This may indicate the long-term effects of antenatal CT on the normalization of newborn weight. A significant decrease in the mean corpuscular volume (MCV) was noted across all the groups of newborns from the mothers with PAS, except one with CT within 2–7 days before delivery. This may indicate the occurrence of microcytic anemia when CT is administered later than seven days before delivery or in the absence of CT. In contrast, CT in the period of 2–7 days before delivery prevented microcytic anemia. Additionally, the PAS group without antenatal CT showed significantly lower mean concentration hemoglobin (MCH) values in their newborns (*p* = 0.01) compared to the control group without CT, which may indicate a direct effect of PAS on the occurrence of iron deficiency anemia in newborns. No significant differences were found between the groups compared in terms of other clinical characteristics.

To determine the relationship between PAS and the levels of hsa-miR-382-5p and hsa-miR-199a-3p in the blood plasma of the pregnant women and their infants, all samples with PAS were divided into groups depending on the depth of PAS—accreta, increta, and percreta. In each of these groups, the following two subgroups were formed: one subgroup included samples with antenatal corticosteroid therapy (CT) more than 14 days before delivery and without RDS prophylaxis at all, since no significant differences in the hsa-miR-382-5p and hsa-miR-199a-3p levels were found between these two sample sets according to the preliminary data which are not presented; the second subgroup included samples with CT 2–7 days before delivery, along with those who had received CT 7–14 days before delivery since no significant differences in two miRNAs levels were found between these two sample sets according to the preliminary data which are not presented. All subgroups with PAS were compared to the control group without PAS and without CT. The “−ΔCt” values were calculated based on the difference between the Ct value of the analyzed miRNA and the Ct value of the exogenous RNA UniSp6.

**Figure 2:**
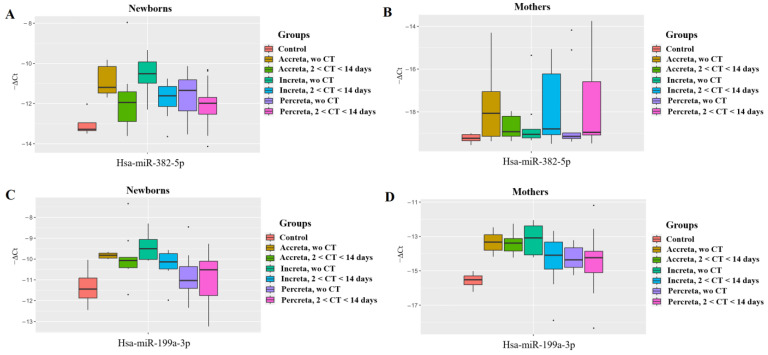
The dependence of hsa-miR-382-5p and hsa-miR-199a-3p content in the blood plasma of newborns and their mothers on the severity of placenta accreta spectrum (PAS) and the timing of antenatal corticosteroid therapy (CT). Levels of miR-382-5p (−∆Ct, PCR data) in the blood plasma of newborns from mothers with placenta accreta or placenta increta or placenta percreta without CT or with CT 2–14 days before delivery in comparison with control group—without PAS and without CT (**A**). Levels of miR-382-5p (−∆Ct, PCR data) in the blood plasma of pregnant women with placenta accreta or placenta increta or placenta percreta without CT or with CT 2–14 days before delivery in comparison with control group—without PAS and without CT (**B**). Levels of miR-199a-3p (−∆Ct, PCR data) in the blood plasma of newborns from mothers with placenta accreta or placenta increta or placenta percreta without CT or with CT 2–14 days before delivery in comparison with control group—without PAS and without CT (**C**). Levels of miR-199a-3p (−∆Ct, PCR data) in the blood plasma of pregnant women with placenta accreta or placenta increta or placenta percreta without CT or with CT 2–14 days before delivery in comparison with control group—without PAS and without CT (**D**). “Wo” means “without”.

In the analysis of the hsa-miR-382-5p levels in neonatal blood plasma (Figure 2A, Table 3), a significant increase was observed in the “accreta without CT” (*p* = 0.006) and “increta without CT” (*p* = 0.005) groups compared to the “Control without CT” group. Additionally, the timing of the antenatal corticosteroid therapy (CT) influenced the hsa-miR-382-5p levels in neonates with placenta accreta or increta. Specifically, the hsa-miR-382-5p levels in the “CT 2–14 days before delivery” groups in the case of accreta or increta were close to that in the “Control without CT” group, suggesting that corticosteroid therapy within this timeframe has a positive effect although the median values in these groups do not reach normal values. It is important to note that no significant differences were detected among the compared groups of newborns from the mothers with placenta percreta.

Similar changes in levels were detected for hsa-miR-199a-3p in neonatal blood plasma (Figure 2C, Table 3), as follows: A significant increase was found in the “accreta without CT” (*p* = 0.006) and “increta without CT” (*p* = 0.005) groups compared to the “Control without CT” group; the hsa-miR-199a-3p levels in the “CT 2–14 days before delivery” groups in the case of accreta or increta were close to that in the “Control without CT” group; no significant differences were found among the groups of neonates from mothers with placenta percreta.

Regarding the analysis of miRNA in maternal blood, no significant changes in hsa-miR-382-5p levels were found while comparing the PAS groups with the control group (Figure 2B, Table 4). In contrast, the analysis of hsa-miR-199a-3p in the blood plasma of mothers revealed a significant increase in its levels across all the PAS groups (accreta, increta, percreta) compared to the “Control without CT” group, regardless of the CT (Figure 2D, Table 4).

**Figure 3:**
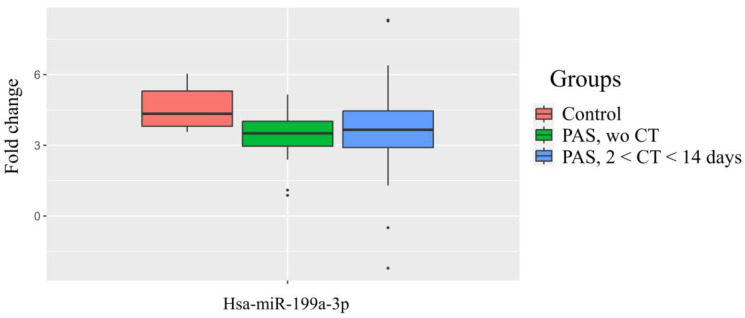
Dynamics of changes in hsa-miR-199a-3p levels in the blood plasma of newborns relative to their mothers’ blood plasma, with and without PAS, depending on the antenatal corticosteroid therapy (CT). “Wo” means “without”.

A notable 3.5–4.6-fold increase in the hsa-miR-199a-3p levels was detected in the neonatal blood plasma compared to the maternal blood plasma (Figure 3, Table 5). Although significant increases in hsa-miR-199a-3p levels were observed in both the neonatal and maternal plasma in cases with PAS (Figure 2C, Table 3, and Figure 2D, Table 4), this increase was more pronounced in the maternal plasma. This is evidenced by the relative decrease in the hsa-miR-199a-3p levels in the neonatal plasma from mothers with PAS compared to the neonates from mothers without PAS (Figure 3).

As indicated in Table 5, the group “PAS without CT” showed significant differences from the control group (*p* = 0.007) in contrast to the comparison of the group with PAS and antenatal CT 2–14 days prior to delivery with control group where no significant differences were found, suggesting a positive effect of antenatal CT.

**Figure 4:**
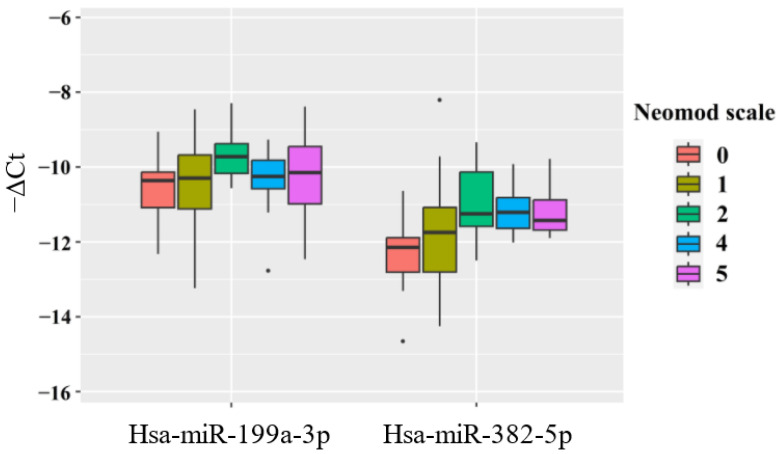
Levels of hsa-miR-199a-3p and hsa-miR-382-5p in the blood plasma of newborns with PAS, categorized by their severity score according to the Neomod scale.

When analyzing newborns with PAS according to the severity score on the Neomod scale—the Neonatal Multiple Organ Dysfunction scale used in neonatal practice in newborns of different gestational age regardless of therapy to assess severity of multiple organ failure for prediction of fatal outcomes—an increase in the hsa-miR-382-5p levels was observed in the blood plasma of newborns with scores of 2, 4, and 5, compared to those with a score of 0 (Figure 4, Table 6) with the significant changes in newborns with Neomod score > 4 (*p* = 0.0096). A similar trend was noted in the quantitative analysis of the hsa-miR-199a-3p levels in the blood plasma of newborns, although this did not reach statistical significance (Table 6).

It is important to highlight that the group with a score of 1 on the Neomod scale included newborns with only moderate respiratory dysfunction. In contrast, the group with a score of 2 included newborns either with severe respiratory dysfunction or with a combination of moderate respiratory dysfunction and moderate dysfunction of the cardiovascular or urinary systems. The groups with scores of 4–5 included newborns experiencing severe respiratory dysfunction combined with moderate dysfunction of the cardiovascular and/or urinary systems and/or acid–base balance.

The significant changes in hsa-miR-382-5p levels in the blood plasma of newborns from mothers with PAS, based on the severity of the condition according to the Neomod scale, indicate a relationship between this miRNA and dysfunctions in the respiratory, cardiovascular, and urinary systems.

Using the nonparametric Spearman’s rank correlation method to assess the strength and significance of a relationship between the quantitative and qualitative features with the assignment of a rank number in ascending or descending order for paired comparison, the study found several significant relationships regarding the levels of specific miRNAs and clinical parameters in newborns and their mothers, as follows:

- A direct correlation between the levels of hsa-miR-382-5p and hsa-miR-199a-3p in the blood plasma of newborns (r = 0.49; *p* < 0.001);
- an inverse correlation between the level of hsa-miR-199a-3p in the blood plasma of mothers and their newborns with the depth of trophoblast invasion (r = −0.46; *p* < 0.001 for mothers and r = −0.29; *p* = 0.028 for newborns);
- an inverse relationship between hsa-miR-382-5p levels in newborns of women with PAS and their weight (r = −0.39; *p* = 0.002);
- a direct relationship between the level of hsa-miR-382-5p in the blood plasma of the newborn and the required fraction of oxygen in the NICU (r = 0.41; *p* = 0.001), duration of stay in the NICU (r = 0.31; *p* = 0.019), and the severity of the newborn’s condition according to the NEOMOD scale (r = 0.36; *p* = 0.005).

In turn, significant correlations were noted between the required oxygen fraction in the NICU for the newborns of mothers with PAS and various hematological parameters, including fetal red blood cell count (r = −0.47; *p* < 0.001), hemoglobin (HGB) (r = −0.37; *p* = 0.003), hematocrit (r = −0.36; *p* = 0.005), and the coefficient of variation of red blood cell distribution width (r = −0.36; *p* = 0.005). Additionally, there were strong correlations with the duration of NICU stay (r = 0.71; *p* = 0), total hospitalization duration (r = 0.49; *p* < 0.001), and the severity of the newborn’s condition according to the NEOMOD scale (r = 0.68; *p* = 0).

**Figure 5:**
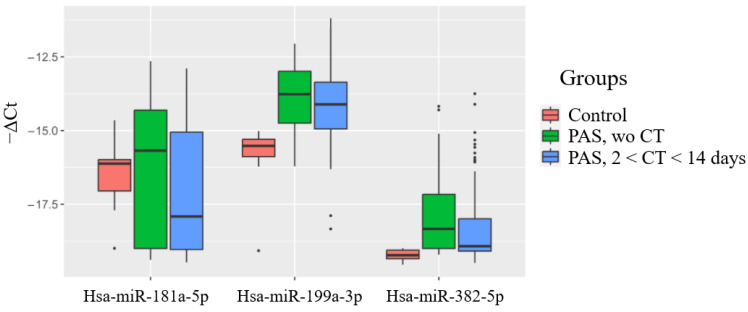
Levels of miR-181a-5p, miR-199a-3p and miR-382-5p in blood plasma of pregnant women with/without PAS and with/without antenatal corticosteroid therapy. “Wo” means “without”.

Based on these correlations, the study aimed to evaluate the potential of using the levels of hsa-miR-199a-3p and hsa-miR-382-5p in the maternal blood plasma to predict neonatal complications. Previous meta-analysis results [37] and our own observations [18] have indicated that newborns whose mothers received antenatal corticosteroids after 34 weeks of gestation had a significantly lower risk of developing RDS and transient tachypnea of the newborn (TTN), along with reduced surfactant and mechanical ventilation use, shorter durations of oxygen supplementation, lower peak inspired oxygen concentrations, shorter NICU stays, and higher Apgar scores than the controls. In this regard, the overall dynamics of changes in the level of hsa-miR-199a-3p and hsa-miR-382-5p in the blood plasma of pregnant women without PAS and in the case of PAS, without subdividing them into their morphological types, in the presence or absence of RDS prophylaxis was assessed (Figure 5). As illustrated in Figure 5, a significant increase in hsa-miR-199a-3p and hsa-miR-382-5p levels was observed across the different PAS groups compared to the control group without PAS (Figure 5, Table 7). We decided to use hsa-miR-181a-5p as a reference endogenous miRNA instead of the exogenous UniSp6 for the quantitative assessment of the hsa-miR-199a-3p and hsa-miR-382-5p levels in the pregnant women’s blood when constructing logistic regression models for the prediction of neonatal complications, since no significant differences in the hsa-miR-181a-5p levels were found among the groups of maternal blood plasma samples (Figure 5, Table 7), and it did not contribute to the separation of the clusters of neonatal plasma samples from the mothers with and without PAS while using the PLS-A method (Figure 1).

**Figure 6:**
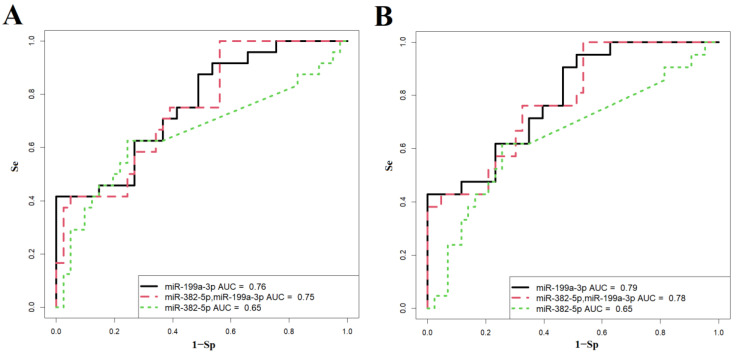
Logistic regression models for predicting neonatal complications by plasma miR-199a-3p and/or miR-382-5p levels in pregnant women with PAS using miR-181a-5p as a reference endogenous RNA. (**A**) Respiratory complications probability models. (**B**) Cardiovascular complications probability models. Se—sensitivity, Sp—specificity.

The probabilities of neonatal complications—specifically respiratory disorders (including RDS, congenital pneumonia, and transient tachypnea) and cardiovascular disorders—were calculated by constructing logistic regression models (see Figure 6A,B) based on quantitative real-time PCR data (−ΔCt values). This analysis assessed the levels of miR-199a-3p and/or miR-382-5p in the blood plasma of pregnant women with PAS, using endogenous RNA miR-181a-5p as a reference. In this context, the dependent variable (response variable) was the presence of neonatal complications, coded as follows: 0 for absence of complications and 1 for presence of complications.

The characteristics of these models are detailed in Table 8. Among the constructed models for predicting respiratory disorders in newborns, Model 2 (shown in Figure 6A) demonstrated the best diagnostic value. It can predict, with 100% sensitivity, the need for invasive mechanical ventilation (IMV) or high-frequency oscillatory ventilation (HFOV) in newborns during the early neonatal period, based on the levels of miR-199a-3p and miR-382-5p in the maternal blood plasma shortly before delivery.

For predicting cardiovascular disorders in newborns, Model 1 (illustrated in Figure 6B) also exhibits strong diagnostic value. This model can predict, with 95% sensitivity (as shown in Table 8), the need for cardiotonic therapy for the newborn in the early neonatal period, based solely on the level of miR-199a-3p in the maternal blood plasma prior to delivery.

**Figure 7:**
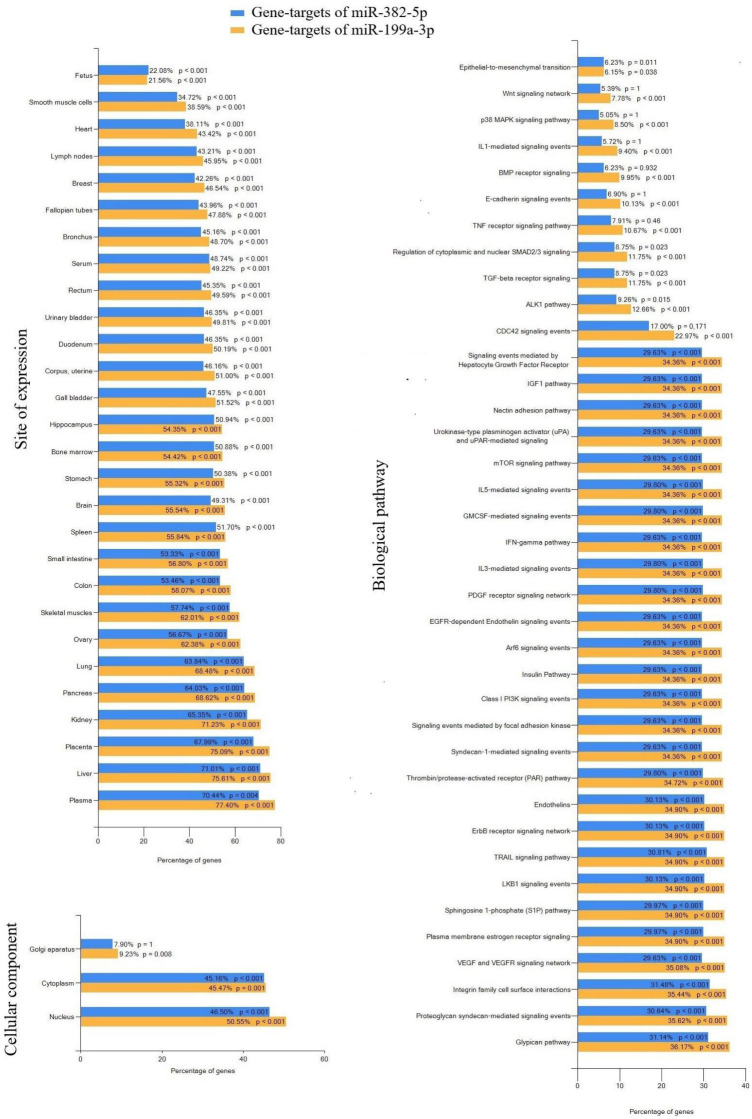
Enrichment analysis of gene targets of hsa-miR-382-5p and hsa-miR-199a-3p using FunRich software tool.

To understand the role of hsa-miR-382-5p and hsa-miR-199a-3p in the pathogenesis of the neonatal complications in the newborns of mothers with PAS, we identified their potential and experimentally validated target genes using the miRTargetLink 2.0 program. This was followed by an analysis of the identified gene sets in the FunRich software tool (Version 3.1.3) for functional enrichment, considering a significance threshold of *p* < 0.05 (Figure 7).

The expression sites of 35–77% of the gene targets for hsa-miR-382-5p and hsa-miR-199a-3p were found across various organs and systems, including the placenta, kidney, lung, heart, uterine corpus, serum, and plasma (Figure 7). In terms of cellular components, 46.50% (*p* < 0.001) of the protein products of the gene targets of hsa-miR-382-5p and 50.55% (*p* < 0.001) of those for hsa-miR-199a-3p were located in the nucleus. Additionally, 45.16% (*p* < 0.001) and 45.47% (*p* = 0.001) of the targets were found in the cytoplasm, while 9.23% (*p* = 0.008) of the targets for hsa-miR-199a-3p were located in the Golgi apparatus (Figure 7).

The significantly enriched pathways associated with the gene targets of these miRNAs included the glypican pathway, which is known to regulate cell growth, motility, and differentiation through fibroblast growth factors (FGFs), vascular endothelial growth factor-A (VEGF-A), transforming growth factor-β (TGF-β), and Wnt signaling [38]; the mTOR (mammalian target of rapamycin) signaling pathway, which controls cell proliferation, migration, cytoskeleton remodeling, ion transport, and glucose metabolism [39]; pathways involved in inflammatory processes, such as sphingosine 1-phosphate (S1P), thrombin/protease-activated receptor (PAR), endothelin, TGF-beta receptor, and IL-1- and IL-3-mediated signaling pathways; Arf6 signaling events, which play a crucial role in innate immunity and host–pathogen interactions [40]; cell death signaling involving TRAIL and TNF receptors; and LKB1 and IGF1 pathways that regulate lipid, cholesterol, and glucose metabolism [41,42]. Additionally, pathways associated with epithelial-to-mesenchymal transition were identified (Figure 7).

## 3. Discussion

While maternal outcomes following pregnancies complicated by PAS are well documented, reports on neonatal outcomes in these cases are limited. Previous retrospective studies consistently indicated high rates of admissions to neonatal intensive care units (NICUs) and a significant need for mechanical ventilation in pregnancies affected by PAS [43]. The primary perinatal complications observed in premature infants born to mothers with PAS in this study included transient tachypnea of the newborn (44%), RDS (12%), congenital pneumonia (41%), congenital anemia (20%), and intraventricular hemorrhage (8%). RDS, which results from a primary deficiency of surfactants and the immaturity of lung tissue due to prematurity, along with congenital pneumonia, can lead to the development of acute respiratory distress syndrome (ARDS) [44]. The mortality rate associated with ARDS remains high, accounting for 30% of all fatalities in intensive care units. [45,46,47]. Morphologically, RDS and ARDS exhibit similar characteristics, including immaturity and antenatal damage to the structures of the air–blood barrier, as well as pneumonia and pulmonary ischemia with the formation of hyaline membranes [44].

Numerous studies have been published on the molecular mechanisms involved in the pathogenesis and pathophysiology of ARDS, many of which were detailed in a review article by Huang Q. et al. [48]. The author summarized that lung barrier dysfunction during ARDS results from the death of alveolar epithelial and pulmonary endothelial cells, which can be triggered by apoptosis pathways such as FasL, TNF-α/TNFR1, and TNF-related apoptosis-inducing ligand (TRAIL) signaling events. Additionally, the article discussed the various signals that regulate inflammatory processes during ARDS, particularly those known to activate the RhoA/ROCK pathway, including IL-1, TGF-β, thrombin, sphingosine-1 phosphate (S1P), and endothelin-1. It also highlighted the factors that alter the activity of the PI3K/AKT pathway through the mammalian target of rapamycin (mTOR) or NF-κB, leading to NLRP3 inflammasome activation or increased levels of inflammatory cytokines. Furthermore, the epithelial–mesenchymal transition (EMT) has been identified as a major factor contributing to epithelial barrier dysfunction and worsening pulmonary edema through the modulation of Wnt signaling in the alveolar epithelium. This process results in the loss of epithelial morphology and the acquisition of mesenchymal characteristics, along with the expression of profibrotic proteins that contribute to pulmonary fibrosis. In this study, we found that these signaling pathways are potentially regulated by two microRNAs, miR-382-5p and miR-199a-3p, (Figure 7) which were significantly elevated in the blood plasma of the day-old neonates and/or their mothers with PAS.

There has been an increasing emphasis on the role of miRNAs in RDS, particularly through their ability to target specific genes to regulate signaling pathways [49,50]. Certain miRNAs play significant roles in the inflammatory response associated with ARDS. For instance, miR-199a-3p has been linked to inflammatory lung diseases, including sepsis-induced ARDS [51]. Notably, this miRNA regulates the synthesis and release of various inflammatory mediators by macrophages [52], which account for nearly half of the immune cells in the lungs [53,54]. Emerging evidence has highlighted the critical role of extracellular vesicles from alveolar macrophages in the inflammatory processes of ARDS, particularly secretory autophagosomes (SAPs) [55]. One of the regulators of SAP secretion is miR-199a-3p, which influences the expression of the target gene PAK4 [52], a serine/threonine kinase identified as a key regulator of TNF-induced microparticle release [56]. Studies have shown that SAPs derived from alveolar macrophages contribute to ARDS through the excessive secretion of IL-1β, which exacerbates inflammation and pathological injury in the lung tissue [55]. Overexpression of miR-199a-3p has been observed in the lungs of mice with ARDS, where the miR-199a-3p antagomir significantly inhibited SAP release, while the miR-199a-3p mimetic promoted SAP release in the bronchoalveolar lavage fluid (BALF), resulting in the alleviation or intensification of LPS-stimulated ARDS, respectively [52]. These results are consistent with findings from this study that noted an increase in hsa-miR-199a-3p levels in the blood plasma of newborns from mothers with PAS. This increase manifests as severe respiratory distress in the early neonatal period, necessitating invasive ventilation or high-frequency ventilation (HFVL).

Another possible pathogenetic mechanism for respiratory disorders in premature infants born to mothers with PAS, particularly concerning the elevated levels of hsa-miR-199a-3p circulating in maternal and fetal blood, is its negative impact on the differentiation of alveolar type II cells, consequently affecting surfactant protein production [57]. The major protein component of pulmonary surfactant SP-A (a product of the SFTPA gene), is developmentally regulated in the fetal lung. It serves as a marker of alveolar type II cell differentiation. Additionally, SP-A plays a vital role in innate immunity by enhancing the uptake and destruction of various pathogens by alveolar macrophages [58,59]. Moreover, it is secreted into the amniotic fluid from the fetal lung, acting as a signaling molecule for the initiation of labor [60,61,62].

During a normal pregnancy, there is a developmental decline in the expression of the miR-199a/-214 cluster in the fetal lung, which leads to the increased expression of key gene targets responsible for alveolar type II cell differentiation and enhanced SP-A expression by term [57]. This dependence of miR-199a/-214 cluster expression on gestational age can be explained by the increased TGF-β signaling during early to mid-gestation, when the fetal lung is relatively hypoxic. This signaling enhances the expression of ZEB1, a transcription factor that stimulates miR-199a/miR-214 cluster expression. As vascularization of the fetal lung increases during the third trimester and near term, heightened oxygen tension leads to decreased TGF-β signaling and repression of ZEB1, resulting in the reduced expression of miR-199a/miR-214. Overexpression of miR-199a-3p, -5p, and miR-214 in human fetal lung epithelial cells has been shown to inhibit SP-A expression and the expression of transcription factors CREB1 and C/EBPβ, which are crucial for fetal lung development [63,64]. Interestingly, ZEB1 is an EMT (epithelial–mesenchymal transition) factor that downregulates epithelial genes while activating mesenchymal genes, promoting a highly invasive cell phenotype [65,66]. This is typical for extravillous trophoblast cells of the placenta in the case of PAS, which exhibit an abnormally aggressive EMT that does not cease at the end of the first trimester but continues throughout the pregnancy [67,68].

Thus, the following mechanism of pathogenesis of respiratory disorders in neonates from mothers with PAS. The elevated level of miR-199a-3p in the maternal blood plasma from the cases with PAS may reflect the excessive EMT of extracellular trophoblasts under chronic inflammatory conditions in the uterine decidua due to endometritis, antecedent curettage, or incompetent uterine scars following a cesarean section. According to Kalluri R. [69], macrophages and activated resident fibroblasts secrete growth factors such as TGF-β, chemokines, and matrix metalloproteinases (MMP-2, -3, -9) in these circumstances. The presence of chorionic villi in the layers of the myometrium results in an abnormal gas exchange in the maternal–fetal system, creating hypoxic conditions for the fetus, including the lung tissue. Under these conditions, TGF-β signaling in lung tissue increases, raising the expression of ZEB1 and, consequently, hsa-miR-199a-3p, leading to immature lung structures and reduced surfactant synthesis. Additionally, we observed elevated levels of hsa-miR-199a-3p in the blood plasma of neonates. As indicated in Figure 3, in cases of PAS, the level of hsa-miR-199a-3p in the maternal blood plasma is higher than that in the neonatal blood plasma, compared to pregnancies without PAS. This represents an additional negative factor influencing the damage to fetal lung tissue due to circulating maternal hsa-miR-199a-3p.

Moreover, this study revealed significant negative correlations between the levels of hsa-miR-199a-3p in the maternal and fetal blood plasma and the severity of PAS; specifically, lower levels of hsa-miR-199a-3p in the maternal and fetal bloodstream are associated with deeper placental invasion into the myometrial layers. This negative relationship can be explained by the increasing intrauterine hypervascularization, with the presence of tortuous anastomosing vessels of a large caliber in the case of the placenta percreta in contrast with placenta accreta or increta, which improves the blood supply and oxygenation of the placenta and, as a sequence, causes a decrease in the hsa-miR-199a-3p expression through decreased TGF-β signaling and the repression of ZEB1 as discussed above.

The elevated level of hsa-miR-382-5p detected in the blood plasma of newborns from mothers with placenta accreta may represent an additional pathogenetic link in the occurrence of neonatal complications. Furthermore, the levels of hsa-miR-199a-3p and hsa-miR-382-5p in the blood plasma of newborns were found to correlate significantly and positively with each other. This correlation may be explained by the presence of a common experimentally validated target gene, PTEN (according to miRTargetLink), which is involved in cell functions including proliferation, migration, and metabolism [70]. Dysregulated PTEN expression has been found in blastocyst implantation [71], preeclampsia [72,73], pulmonary diseases [74], and PAS [75]. Localized primarily in the syncytiotrophoblast (STB), endothelial cells surrounding fetal blood vessels, and to a lesser extent in the stroma of normal placenta [75], increased expression of PTEN impairs human trophoblast cell invasion and is associated with the development of preeclampsia [76]. In contrast, PTEN mRNA and protein levels are reduced in the placenta tissue affected by PAS compared to normal placenta [75], suggesting its critical role during pregnancy.

It is known that miR-382-5p is a member of the chromosome 14 miRNA cluster (C14MC), which is one of the largest clusters of pregnancy-related miRNAs, comprising 52 miRNAs [27]. This cluster is involved in embryonic development, endothelial cell migration, and angiogenesis during placental development [77]. miR-382-5p, as an ortholog of the C14MC found in equines, has been shown to be enriched in the blood serum of pregnant mares compared to non-pregnant mares [78]. Additionally, the aberrant expression of miR-382-5p in rat lung tissues has been reported as a potential cause of bronchopulmonary dysplasia (BRD) through the suppression of M1 macrophage polarization [79,80].

Regarding the regulation of macrophage function, miR-382-5p may play a significant role in the pathogenesis of ARDS, as macrophages are a crucial component of pulmonary innate immunity, comprising nearly half of the immune cells in the lungs, and the balance between M1 and M2 macrophage phenotypes influences the various stages of ARDS [81] [82,83,84,85]. In the acute exudative phase of ARDS, macrophages are predominantly M1-polarized, releasing pro-inflammatory factors that induce a severe inflammatory response. In the later stages of ARDS, macrophages mainly adopt an M2-polarized phenotype, which can lead to pathological fibroplasia and pulmonary fibrosis.

Mechanisms regulating macrophage function involving miR-382-5p have been demonstrated using microglial cells, which are resident macrophages in the central nervous system that perform immune surveillance in the brain and spinal cord [86]. Through the upregulation of Circ_0006640, which can directly sequester miR-382-5p, and the elevation of IGF1, a target of miR-382-5p, the microglial cells showed protection from LPS-induced apoptotic, inflammatory, and oxidative injuries. IGF-1 is a major growth hormone critical for prenatal lung growth and organogenesis [87]. Local synthesis of IGF-1 in lung tissue occurs in type II pneumocytes, alveolar macrophages, and mesenchymal cells. In animal models, mutations in the IGF-1 gene disrupt the architecture of lung tissue, leading to atelectatic lungs, respiratory failure, and high postnatal mortality.

In our study, the level of hsa-miR-382-5p in the blood plasma of premature infants born to mothers with PAS was significantly higher in cases where antenatal prophylaxis for RDS was absent or implemented more than 14 days before delivery, compared to premature infants born to mothers without PAS and without antenatal prophylaxis for RDS. The level of hsa-miR-382-5p in the blood plasma of newborns from mothers with PAS tended to normalize after antenatal prophylaxis for RDS 2–14 days before delivery and did not significantly differ from the levels in the blood plasma of newborns from the mothers without PAS. A markedly increased level of hsa-miR-382-5p in the blood plasma of premature infants from the mothers with PAS, particularly in the absence of antenatal prophylaxis for RDS or when implemented more than 14 days before delivery, likely caused a decrease in IGF-1 across various organs and tissues of the newborn, including the lungs. This decrease helps explain the presence of respiratory disorders in this group of patients, as well as the statistically significant correlations between the level of hsa-miR-382-5p in the blood plasma of the newborn and factors such as weight (r = −0.39; *p* = 0.0027), required oxygen fraction in the NICU (r = 0.41; *p* = 0.0016), length of stay in the NICU (r = 0.31; *p* = 0.019), and severity of the newborn’s condition according to the NEOMOD scale (r = 0.36; *p* = 0.0051).

In addition to respiratory support, newborns from mothers with PAS require cardiotonic therapy due to cardiovascular dysfunction. It was found that miRNAs derived from the precursor miR-199a play a key role in maintaining cardiac homeostasis, particularly through the regulation of endothelial nitric oxide synthase (eNOS) in the endothelium [88,89,90]. A common mechanism underlying many cardiovascular diseases is endothelial dysfunction, which is characterized by the reduced availability of nitric oxide (NO) [91]. It has been demonstrated that the inhibition of miR-199a-3p enhances eNOS activity and decreases the degradation of NO, thereby increasing its bioavailability and modulating vascular contractility [90].

Given the relationships identified in this study between the levels of hsa-miR-199a-3p and hsa-miR-382-5p in the blood plasma of pregnant women and their newborns, as well as the severity of respiratory and cardiac disorders during the neonatal period, we constructed logistic regression models to predict these disorders. These models take into account the established roles of these miRNAs in surfactant synthesis by alveolar cells, fetal organogenesis, the formation of proper lung tissue architecture, and the regulation of the cardiovascular system as reported in the literature. The models developed in this study allow for the prediction of the need for cardiotonic therapy and invasive mechanical ventilation (IMV) or high-frequency oscillatory ventilation (HFOV) for newborns in the early neonatal period, with a sensitivity of 95–100%. However, the implementation of these models in clinical practice will require large-scale studies using independent test samples.

## 4. Materials and Methods

### 4.1. Patients

All patients included in the study were admitted to the National Medical Research Center for Obstetrics, Gynecology, and Perinatology, named after Academician V.I. Kulakov of the Ministry of Healthcare of the Russian Federation, for pregnancy and delivery management. They signed an informed consent to participate, and the study was approved by the ethics committee of the center.

In the main group (*n* = 69), all the women underwent an operative delivery via cesarean section due to PAS. In 66 cases, the delivery was planned, while 3 cases required an emergency cesarean section due to bleeding.

In the control group (*n* = 11), all the women also underwent cesarean sections. In 2 cases, the procedure was planned, with the indications being preeclampsia in 1 case and threatened preterm labor in the other. A total of 9 women required emergency cesarean sections for various reasons including bleeding (1 case), onset of labor (2 cases), fetal condition deterioration (3 cases), preeclampsia (1 case), suspected uterine scar failure (1 case), and maternal somatic pathology (1 case).

Antenatal prophylaxis for RDS was conducted following current clinical guidelines for preterm labor management. The drug “dexamethasone” (manufacturer “Ellara”, Russia, Pokrov) was administered intramuscularly at a dose of 8 mg three times, with an 8 h interval between doses (total dose: 24 mg).

### 4.2. Isolation of RNA from Peripheral Blood Plasma Samples

Peripheral blood samples were collected into VACUETTE^®^ EDTA tubes and centrifuged for 20 min at 300× *g* at 4 °C. Plasma was then collected and centrifuged again for 10 min at 16,000× *g*. RNA was isolated from 200 μL of plasma using the miRNeasy Serum/Plasma kit (Qiagen, Hilden, Germany).

### 4.3. Deep Sequencing of miRNA

cDNA libraries were synthesized using 6 μL of total RNA eluate from the neonatal plasma samples with the NEBNext^®^ Multiplex Small RNA Library Prep Set for Illumina^®^ (Set2, New England Biolab^®^, Frankfurt am Main, Germany, cat. No. E7580S), following the manufacturer’s protocol. The cDNA libraries were amplified and purified using 6% polyacrylamide gel, with the 140–160 base pair fraction extracted. The quantity and quality of the cDNA libraries were assessed with an Agilent 2100 Bioanalyzer (Agilent Technologies, Waldbronn, Germany) using the High Sensitivity DNA reagents kit (Agilent Technologies, Santa Clara, CA, USA). Sequencing of the cDNA libraries was performed on the NextSeq 500 platform (Illumina, San Diego, CA, USA, cat. no. SY-415-1001), following the manufacturer’s instructions. For sequence annotation, the GRCh38.p15 and miRBase v21 databases were utilized, along with the STAR RNAseq aligner program. The DESeq2 software package 1.42.0 was used to normalize the cDNA read counts in each sample.

### 4.4. Reverse Transcription and Quantitative Real-Time PCR

First, 5 µL of the 14 µL eluate obtained from the miRNeasy Serum/Plasma Kit column (Qiagen, Hilden, Germany), which contained plasma RNA, was used for cDNA synthesis with the miRCURY LNA RT Kit (Qiagen, Hilden, Germany), following the manufacturer’s protocol. Quantitative real-time PCR was then carried out using the miRCURY LNA SYBR Green PCR Kit (Qiagen, Hilden, Germany) and miRNA-specific primers (miRCURY LNA miRNA PCR Assay, Qiagen, Hilden, Germany), according to the manufacturer’s instructions, using a StepOnePlus™ thermal cycler (Applied Biosystems, Waltham, MA, USA). Relative miRNA expression in plasma was calculated using the ∆Ct method, with UniSp6 serving as the reference RNA.

### 4.5. Statistical Data Processing

Scripts written in R 4.3.2 [92] and the RStudio software, version 2023.09.1 [93], were used for statistical analysis. The Shapiro–Wilk test was applied to assess the normality of the data. For non-normally distributed data, paired comparisons were made using the Mann–Whitney test. Variables that did not follow a normal distribution were described as the median (Me) and quartiles Q1 and Q3 in the format Me (Q1; Q3). A significance threshold of *p* = 0.05 was set in pairwise comparison, and if the *p*-value was less than 0.001, it was indicated as *p* < 0.001. For multiple comparisons, a lower critical significance level was calculated using the following formula: *p* = (1 − 0.95)/n, where n is the number of comparisons made.

The logistic regression models were developed in RStudio through the stepwise inclusion and exclusion of miRNA marker molecules based on their contribution to the model. The predictive performance of the model was evaluated using ROC (Receiver Operating Characteristic) analysis, assessing the AUC (Area Under the Curve), statistical significance, specificity, and sensitivity.

## Figures and Tables

**Table 1:** Significant changes in the level of miRNA in the blood plasma of newborns from mothers without PAS and without RDS prophylaxis compared to newborns from mothers with PAS and RDS prophylaxis more than 14 days before delivery.

	miRNA	BaseMean	log2FoldChange	lfcSE*	*p*-Value
1	hsa-miR-215-5p	98.7	5.8	1.2	4.2 × 10^−6^
2	hsa-miR-516b-5p	215.1	5.2	1.1	6.8 × 10^−6^
3	hsa-miR-182-5p	55.2	4.7	1.1	2.0 × 10^−5^
4	hsa-miR-183-5p	143.4	4.1	1.0	6.6 × 10^−5^
5	hsa-miR-192-5p	503.9	1.6	0.4	<0.001
6	hsa-miR-1323	30.6	3.8	1.1	0.001
7	hsa-miR-760	15.0	−3.2	1.0	0.001
8	hsa-let-7f-5p	992.7	2.2	0.7	0.002
9	hsa-miR-26a-5p	1195.9	1.7	0.6	0.003
10	hsa-miR-199a-3p	320.7	−1.8	0.6	0.004
11	hsa-miR-200c-3p	121.4	−4.1	1.4	0.004
12	hsa-miR-199b-3p	160.3	−1.7	0.6	0.004
13	hsa-let-7g-5p	1207.6	1.8	0.6	0.005
14	hsa-miR-10a-5p	1121.6	2.7	1.0	0.006
15	hsa-miR-146b-5p	130.2	1.4	0.5	0.007
16	hsa-miR-99b-3p	8.9	−3.4	1.2	0.008
17	hsa-miR-218-5p	9.3	−4.0	1.6	0.011
18	hsa-miR-150-5p	24.7	1.4	0.6	0.019
19	hsa-miR-29a-3p	35.6	1.9	0.8	0.021
20	hsa-miR-181b-5p	124.9	−2.3	1.0	0.028
21	hsa-miR-378c	8.8	1.8	0.8	0.029
22	hsa-miR-26b-5p	102.9	1.2	0.5	0.029
23	hsa-miR-30e-3p	45.6	1.5	0.7	0.031
24	hsa-miR-483-3p	37.4	2.1	0.9	0.032
25	hsa-miR-194-5p	209.4	1.6	0.7	0.033
26	hsa-miR-99a-5p	1362.0	−1.4	0.7	0.037
27	hsa-miR-2110	38.6	−1.9	0.9	0.038
28	hsa-let-7d-3p	244.3	1.2	0.6	0.041
29	hsa-miR-382-5p	125.1	−2.2	1.2	0.045

*lfcSE—the standard error estimate for the log_2_ fold change estimate.

**Table 2:** Clinical characteristics of premature infants in mothers with and without antenatal corticosteroid therapy (CT), along with comparisons of the corresponding groups using the Wilcoxon-Mann-Whitney U test.

Clinical Parameters	Control, Without CT (*n* = 11), I Group	PAS, Without CT (*n* = 10), II Group	PAS, CT More Than 14 Days Before Delivery (*n* = 13), III Group	PAS, CT During 7–14 Days Before Delivery (*n* = 25), IV Group	PAS, CT During 2–7 Days Before Delivery (*n* = 21), V Group	Wilcoxon–Mann–Whitney U Test, *p*-Value			
						I Group vs. II Group	I Group vs. III Group	I Group vs. IV Group	I Group vs. V Group
Weight of newborn, g	2250.0 (1965.0; 2437.5)	2795.5 (2542.0; 3042.2)	2520.0 (2390.0; 2652.0)	2863.0 (2780.0; 3030.0)	2850.0 (2730.0; 2960.0)	0.001	0.089	<0.001	0.001
Apgar score, 1 min	8.0 (7.0; 8.0)	7.0 (7.0; 8.0)	8.0 (7.0; 8.0)	8.0 (7.0; 8.0)	8.0 (7.0; 8.0)	0.205	0.702	0.606	0.973
Apgar score, 5 min	8.0 (8.0; 9.0)	8.0 (8.0; 8.0)	8.0 (8.0; 8.0)	8.0 (8.0; 9.0)	8.0 (8.0; 9.0)	0.084	0.067	0.425	0.447
WBC	11.4 (9.7; 12.6)	12.2 (9.9; 18.0)	10.4 (9.3; 13.3)	14.1 (9.5; 16.9)	13.2 (10.6; 16.5)	0.417	0.757	0.207	0.189
ACHN	4225.0 (3806.5; 4561.0)	4776.5 (3236.2; 8941.0)	3872.0 (3448.0; 5440.0)	5664.0 (4323.0; 7874.0)	6190.0 (4131.0; 7722.0)	0.475	0.937	0.148	0.155
Ni	0.07 (0.04; 0.08)	0.05 (0.02; 0.11)	0.06 (0.03; 0.09)	0.07 (0.03; 0.11)	0.06 (0.05; 0.09)	0.659	0.781	0.714	0.979
RBC	4.5 (4.3; 4.8)	4.7 (4.1; 4.9)	4.7 (4.4; 4.8)	4.4 (4.0; 4.8)	4.6 (4.4; 4.8)	1.000	0.938	0.48	0.75
RDW-CV	16.0 (15.3; 17.2)	15.7 (15.2; 16.2)	15.8 (15.4; 16.6)	15.8 (15.4; 16.1)	15.8 (15.3; 16.5)	0.769	0.721	0.437	0.652
RDW-SD	63.1 (61.9; 67.9)	57.4 (51.8; 59.3)	58.8 (55.9; 60.4)	58.9 (56.7; 59.7)	60.1 (57.7; 62.9)	0.007	0.047	0.009	0.08
MCV	105.8 (105.0; 108.3)	98.0 (95.3; 102.1)	101.4 (99.4; 103.2)	102.2 (98.5; 103.3)	101.9 (100.4; 105.6)	0.001	0.008	0.002	0.027
HGB, g/L	163.0 (155.5; 180.5)	161.0 (145.5; 167.7)	168.0 (158.0; 179.0)	158.0 (146.0; 173.0)	168.0 (161.0; 171.0)	0.806	0.936	0.583	0.121
MCH	36.6 (35.8; 38.2)	35.0 (34.0; 35.4)	36.2 (35.2; 36.7)	35.5 (35.1; 36.5)	35.9 (35.1; 36.6)	0.010	0.427	0.068	0.185
MCHC	34.6 (34.5; 34.9)	35.4 (35.0; 36.2)	35.7 (35.2; 36.1)	35.4 (35.0; 35.7)	35.1 (34.6; 35.6)	0.050	0.039	0.079	0.287
HTC	47.3 (45.1; 52.1)	42.7 (40.0; 49.5)	47.2 (45.1; 49.8)	44.8 (41.2; 50.6)	47.7 (46.4; 48.9)	0.130	0.606	0.171	0.958
Platelets	324.0 (288.0; 356.0)	323.0 (280.2; 399.0)	281.0 (224.0; 335.0)	354.0 (317.0; 402.0)	339.0 (296.0; 413.0)	0.696	0.428	0.092	0.533
MPV	9.7 (9.0; 9.9)	9.4 (9.2; 9.6)	9.8 (9.4; 10.0)	9.5 (8.9; 10.0)	9.6 (9; 10.1)	0.302	0.720	1.000	0.811
PTC	0.3 (0.2; 0.3)	0.3 (0.2; 0.3)	0.2 (0.2; 0.3)	0.3 (0.3; 0.4)	0.3 (0.2; 0.4)	0.883	0.341	0.283	0.594
PDW	10.4 (9.5; 10.5)	9.7 (8.9; 10.8)	10.2 (9.5; 10.7)	9.1 (8.6; 10.0)	9.8 (9; 10.1)	0.807	0.873	0.273	0.381
PLCR	22.3 (17.6; 24.0)	19.9 (18.5; 23.1)	22.8 (19.2; 24.5)	19.7 (15.9; 24.2)	21.0 (17.8; 25.1)	0.660	0.751	0.789	1.000
DHR	2.0 (1.0; 4.0)	4.5 (3.0; 6.0)	5.0 (2.0; 6.0)	2.0 (2.0; 4.0)	2.0 (2.0; 3.0)	0.115	0.118	0.591	0.978
HD	13.0 (9.0; 14.5)	10.0 (8.0; 14.0)	11.0 (11.0; 13.0)	10.0 (7.0; 15.0)	9.0 (7.0; 11.0)	0.305	0.937	0.315	0.770

PAS, placenta accreta spectrum; CT, corticosteroid therapy; WBC, white blood cells; ACHN, absolute neutrophil count; Ni, neutrophil index; RBC, red blood cells; RDW-CV, RBC distribution width, the coefficient of variation; RDW-SD, RBC distribution width, standard deviation; MCV, mean corpuscular volume; MCH, mean concentration hemoglobin; MCHC, mean corpuscular hemoglobin concentration; HTC, hematocrit; MPV, mean platelets volume; PTC, thrombocrit; PDW, platelet distribution width; PLCR, percentage of giant (˃12 µm) platelets (%); DHR, length of stay in the NICU, days; HD, duration of hospitalization, days. The clinical blood test data and the weight of the day-old newborn are presented as the median (Me) and quartiles Q1 and Q3 in the format: Me (Q1; Q3). The differences between the groups were considered significant when *p* ≤ 0.0125 (see Section 4).

**Table 3:** Quantitative RT-PCR data assessing hsa-miR-382-5p and hsa-miR-199a-3p levels in the blood plasma of newborns from mothers without PAS in the absence of antenatal corticosteroid therapy (CT), as well as from mothers with PAS without or with CT.

miR-382-5p			
ID Group	Group Name	RT-PCR Data	Control Group (1) vs. Groups (2–7)
		Me (Q1; Q3)	Wilcoxon-Mann-Whitney U Test, *p*-Value *
1	Control, wo CT	−13.2 (−13.3; −12.9)	1.000
2	Accreta, wo CT	−11.1 (−11.4; −10.1)	0.006
3	Accreta, 2 < CT < 14 days	−11.9 (−12.8; −11.4)	0.148
4	Increta, wo CT	−10.5 (−10.9; −9.9)	0.005
5	Increta, 2 < CT < 14 days	−11.6 (−12.1; −11.1)	0.036
6	Percreta, wo CT	−11.3 (−12.3; −10.8)	0.075
7	Percreta, 2 < CT < 14 days	−11.9 (−12.5; −11.6)	0.061
**miR-199a-3p**			
1	Control, wo CT	−11.4 (−11.8; −10.9)	1.000
2	Accreta, wo CT	−9.8 (−9.9; −9.7)	0.006
3	Accreta, 2 < CT < 14 days	−10.0 (−10.4; −9.9)	0.106
4	Increta, wo CT	−9.5 (−10.0; −9.0)	0.005
5	Increta, 2 < CT < 14 days	−10.1 (−10.4; −9.7)	0.062
6	Percreta, wo CT	−11.0 (−11.4; −10.3)	0.330
7	Percreta, 2 < CT < 14 days	−10.5 (−11.7; −10.1)	0.470

* The differences between the groups were considered significant when *p* ≤ 0.0083 (see Section 4). “Wo” means “without”. RT-PCR data are presented as the median (Me) and quartiles Q1 and Q3 in the format Me (Q1; Q3).

**Table 4:** The quantitative RT-PCR data for evaluating hsa-miR-382-5p and hsa-miR-199a-3p levels in the blood plasma of pregnant women without PAS in the absence of antenatal CT, in mothers with PAS with or without CT.

miR-382-5p			
ID Group	Group Name	RT-PCR Data	Control Group (1) vs. Groups (2–7)
		Me (Q1; Q3)	Wilcoxon-Mann-Whitney U test, *p*-Value *
1	Control, wo CT	−19.2 (−19.3; −19.0)	1.000
2	Accreta, wo CT	−18.0 (−19.1; −17.0)	0.180
3	Accreta, 2 < CT < 14 days	−18.9 (−19.1; −18.2)	0.070
4	Increta, wo CT	−19.0 (−19.2; −18.8)	0.110
5	Increta, 2 < CT < 14 days	−18.7 (−19.0; −16.2)	0.020
6	Percreta, wo CT	−19.1 (−19.2; −18.9)	0.470
7	Percreta, 2 < CT < 14 days	−18.9 (−19.0; −16.5)	0.024
**miR-199a-3p**			
1	Control, wo CT	−15.5 (−15.8; −15.3)	1.000
2	Accreta, wo CT	−13.3 (−13.8; −12.9)	<0.001
3	Accreta, 2 < CT < 14 days	−13.3 (−13.8; −13.1)	<0.001
4	Increta, wo CT	−13.0 (−14.0; −12.3)	<0.001
5	Increta, 2 < CT < 14 days	−14.0 (−14.9; −13.3)	0.002
6	Percreta, wo CT	−14.3 (−14.8; −13.6)	<0.001
7	Percreta, 2 < CT < 14 days	−14.2 (−15.1; −13.8)	0.015

* The differences between the groups were considered significant when *p* ≤ 0.0083 (see Section 4). “Wo” means “without”. RT-PCR data are presented as the median (Me) and quartiles Q1 and Q3 in the format Me (Q1; Q3).

**Table 5:** Relative content of hsa-miR-199a-3p in the blood plasma of newborns from pregnant women without PAS in the absence of corticosteroid therapy, as well as in the blood plasma of newborns from pregnant women with PAS with or without CT.

miR-199a-3p			Control Group (1) vs. Groups (2,3)
ID Group	Group Name	Me (Q1; Q3)	*p*-Value *
1	Control, wo CT	4.3 (3.8; 5.3)	1.000
2	PAS, wo CT	3.5 (2.9; 4.0)	0.007
3	PAS, 2 < CT < 14 days	3.6 (2.9; 4.4)	0.122

* The differences between the groups were considered significant when *p* ≤ 0.025 (see Section 4). “Wo” means “without”. Fold change values are presented as the median (Me) and quartiles Q1 and Q3 in the format Me (Q1; Q3).

**Table 6:** Comparison of newborns groups from mothers with PAS based on the levels of hsa-miR-199a-3p and hsa-miR-382-5p relative to their scores on the Neomod scale.

	miR-382-5p				miR-199a-3p			
	RT-PCR Data, −ΔCt			*p*-Value *, Mann-Whitney U Test	RT-PCR Data, −ΔCt			*p*-Value *, Mann-Whitney U Test
Groups According to the Neomod Scale	Me	Q1	Q3	Neomod, 0	Me	Q1	Q3	Neomod, 0
Neomod, 0	−12.1	−12.8	−11.8	1.000	−10.3	−11.0	−10.1	1.000
Neomod, 1	−11.7	−12.8	−11.0	0.251	−10.3	−11.1	−9.6	0.672
Neomod, 2	−11.2	−11.5	−10.1	0.073	−9.7	−10.1	−9.3	0.180
Neomod, 4	−11.2	−11.6	−10.8	0.013	−10.2	−10.5	−9.8	0.886
Neomod, 5	−11.4	−11.6	−10.8	0.050	−10.1	−10.9	−9.4	0.927
Neomod, >4	−11.2	−11.6	−10.8	0.009	−10.2	−10.5	−9.8	0.855

* The differences between the groups were considered significant when *p* ≤ 0.01 (see Section 4). RT-PCR data are presented as the median (Me) and quartiles Q1 and Q3.

**Table 7:** Comparison of the hsa-miR-382-5p, hsa-miR-199a-3p and hsa-miR-181a-5p levels in the maternal blood plasma depending on the presence of PAS and corticosteroid therapy (CT).

Group		miR-181a-5p		miR-199a-3p		miR-382-5p	
ID Group	Group Name	Me (Q1; Q3)	Control Group (1) vs. Groups (2,3), *p*-Value *	Me (Q1; Q3)	Control Group (1) vs. Groups (2,3), *p*-Value *	Me (Q1; Q3)	Control Group (1) vs. Groups (2,3), *p*-Value *
1	Control, wo CT	−16.1 (−17.0; −15.9)	1.000	−15.5 (−15.8; −15.3)	1.000	−19.2 (−19.3; −19.0)	1.000
2	PAS, wo CT	−15.6 (−19.0; −14.3)	0.340	−13.7 (−14.7; −12.9)	<0.001	−18.3 (−19.0; −17.1)	<0.001
3	PAS, 2 < CT < 14 days	−17.9 (−19.0; −15.0)	0.690	−14.1 (−14.9; −13.3)	<0.001	−18.9 (−19.0; −17.9)	0.011

* The differences between the groups were considered significant when *p* ≤ 0.025 (see Section 4). “Wo” means “without”. RT-PCR data are presented as the median (Me) and quartiles Q1 and Q3 in the format Me (Q1; Q3).

**Table 8:** Parameters of the logistic regression models presented in Figure 6A,B.

Figure 6A	Wald	*p*-Value	Coefficients	Threshold	Sensitivity	Specificity
1 Model				0.642	0.42	1.00
(Intercept)	1.879	0.060	0.974			
miR-199a-3p	−3.281	0.001	−0.548			
2 Model				0.202	1.00	0.44
(Intercept)	1.706	0.088	1.540			
miR-382-5p	0.796	0.426	0.119			
miR-199a-3p	−2.662	0.008	−0.699			
3 Model				0.422	0.63	0.76
(Intercept)	−2.616	0.009	−0.804			
miR-382-5p	−2.049	0.040	−0.206			
**Figure 6B**	**Wald**	***p*** **-Value**	**Coefficients**	**Threshold**	**Sensitivity**	**Specificity**
1 Model				0.160	0.95	0.49
(Intercept)	1.887	0.050	1.046			
miR-199a-3p	−3.473	0.001	−0.635			
2 Model				0.150	1.00	0.47
(Intercept)	2.005	0.045	2.127			
miR-382-5p	1.282	0.200	0.217			
miR-199a-3p	−2.940	0.003	−0.924			
3 Model				0.380	0.62	0.74
(Intercept)	−3.092	0.002	−1.002			
miR-382-5p	−2.031	0.042	−0.217			

## Data Availability

Data are contained within the article.

## References

[1] Tantbirojn P., Crum C.P., Parast M.M. (2008). Pathophysiology of Placenta Creta: The Role of Decidua and Extravillous Trophoblast. Placenta.

[2] American College of Obstetricians and Gynecologists, and Society for Maternal-Fetal Medicine (2018). Obstetric Care Consensus No. 7: Placenta Accreta Spectrum. Obstet. Gynecol..

[3] Jauniaux E., Bunce C., Grønbeck L., Langhoff-Roos J. (2019). Prevalence and Main Outcomes of Placenta Accreta Spectrum: A Systematic Review and Meta-Analysis. Am. J. Obstet. Gynecol..

[4] Hull A.D., Moore T.R. (2011). Multiple Repeat Cesareans and the Threat of Placenta Accreta: Incidence, Diagnosis, Management. Clin. Perinatol..

[5] Baldwin H.J., Patterson J.A., Nippita T.A., Torvaldsen S., Ibiebele I., Simpson J.M., Ford J.B. (2018). Antecedents of Abnormally Invasive Placenta in Primiparous Women: Risk Associated with Gynecologic Procedures. Obstet. Gynecol..

[6] Wu S., Kocherginsky M., Hibbard J.U. (2005). Abnormal Placentation: Twenty-Year Analysis. Am. J. Obstet. Gynecol..

[7] Spillane N.T., Zamudio S., Alvarez-Perez J., Andrews T., Nyirenda T., Alvarez M., Al-Khan A. (2018). Increased Incidence of Respiratory Distress Syndrome in Neonates of Mothers with Abnormally Invasive Placentation. PLoS ONE.

[8] Munoz J.L., Kimura A.M., Julia J., Tunnell C., Hernandez B., Curbelo J., Ramsey P.S., Ireland K.E. (2022). Impact of Placenta Accreta Spectrum (PAS) Pathology on Neonatal Respiratory Outcomes in Cesarean Hysterectomies. J. Matern.-Fetal Neonatal Med..

[9] Haram K., Mortensen J.H.S., Wollen A.-L. (2003). Preterm Delivery: An Overview. Acta Obstet. Gynecol. Scand..

[10] Patel R.M. (2016). Short- and Long-Term Outcomes for Extremely Preterm Infants. Am. J. Perinatol..

[11] Doyle L.W. (2001). Outcome at 5 Years of Age of Children 23 to 27 Weeks’ Gestation: Refining the Prognosis. Pediatrics.

[12] Saigal S., Doyle L.W. (2008). An Overview of Mortality and Sequelae of Preterm Birth from Infancy to Adulthood. Lancet.

[13] Balashova E.N., Ionov O.V., Kirtbaya A.R., Nikonets A.D., Mikheeva A.A., Vasilchenko O.N., Zubkov V.V., Shmakov R.G., Degtyarev D.N. (2021). The Features of Respiratory and Cardiovascular Disorders in Preterm Infants Born to Mothers with Abnormally Invasive Placenta. Obstet. Gynegology.

[14] Jobe A.H., Soll R.F. (2004). Choice and Dose of Corticosteroid for Antenatal Treatments. Am. J. Obstet. Gynecol..

[15] Gyamfi-Bannerman C., Thom E.A., Blackwell S.C., Tita A.T.N., Reddy U.M., Saade G.R., Rouse D.J., McKenna D.S., Clark E.A.S., Thorp J.M.J. (2016). Antenatal Betamethasone for Women at Risk for Late Preterm Delivery. N. Engl. J. Med..

[16] Ballard P.L., Ballard R.A. (1995). Scientific Basis and Therapeutic Regimens for Use of Antenatal Glucocorticoids. Am. J. Obstet. Gynecol..

[17] Williams M.J., Ramson J.A., Brownfoot F.C. (2022). Different Corticosteroids and Regimens for Accelerating Fetal Lung Maturation for Babies at Risk of Preterm Birth. Cochrane Database Syst. Rev..

[18] Nikonets A.D., Balashova E.N., Ionov O.V., Kirtbaya A.R., Zubkov V.V., Shmakov R.G., Degtyarev D.N. (2024). Prevention of Respiratory Disorders in Late Preterm Neonates Born to Mothers with Abnormally Invasive Placenta. Obstet. Gynegology.

[19] Timofeeva A.V., Fedorov I.S., Suhova Y.V., Tarasova A.M., Ezhova L.S., Zabelina T.M., Vasilchenko O.N., Ivanets T.Y., Sukhikh G.T. (2024). Diagnostic Role of Cell-Free MiRNAs in Identifying Placenta Accreta Spectrum during First-Trimester Screening. Int. J. Mol. Sci..

[20] Timofeeva A.V., Fedorov I.S., Pirogova M.M., Vasilchenko O.N., Chagovets V.V., Ezhova L.S., Zabelina T.M., Shmakov R.G., Sukhikh G.T. (2021). Clusterin and Its Potential Regulatory MicroRNAs as a Part of Secretome for the Diagnosis of Abnormally Invasive Placenta: Accreta, Increta, and Percreta Cases. Life.

[21] Bartel D.P. (2004). MicroRNAs: Genomics, Biogenesis, Mechanism, and Function. Cell.

[22] Alles J., Fehlmann T., Fischer U., Backes C., Galata V., Minet M., Hart M., Abu-Halima M., Grässer F.A., Lenhof H.-P. (2019). An Estimate of the Total Number of True Human MiRNAs. Nucleic Acids Res..

[23] Bartel D.P. (2018). Metazoan MicroRNAs. Cell.

[24] Gebert L.F.R., MacRae I.J. (2019). Regulation of MicroRNA Function in Animals. Nat. Rev. Mol. Cell Biol..

[25] Nakanishi K. (2022). Anatomy of Four Human Argonaute Proteins. Nucleic Acids Res..

[26] Kloosterman W.P., Plasterk R.H.A. (2006). The Diverse Functions of MicroRNAs in Animal Development and Disease. Dev. Cell.

[27] Morales-Prieto D.M., Ospina-Prieto S., Chaiwangyen W., Schoenleben M., Markert U.R. (2013). Pregnancy-Associated MiRNA-Clusters. J. Reprod. Immunol..

[28] Reza A.M.M.T., Choi Y.-J., Han S.G., Song H., Park C., Hong K., Kim J.-H. (2019). Roles of MicroRNAs in Mammalian Reproduction: From the Commitment of Germ Cells to Peri-Implantation Embryos. Biol. Rev. Camb. Philos. Soc..

[29] Hayder H., O’Brien J., Nadeem U., Peng C. (2018). MicroRNAs: Crucial Regulators of Placental Development. Reproduction.

[30] Timofeeva A.V., Fedorov I.S., Shamina M.A., Chagovets V.V., Makarova N.P., Kalinina E.A., Nazarenko T.A., Sukhikh G.T. (2021). Clinical Relevance of Secreted Small Noncoding RNAs in an Embryo Implantation Potential Prediction at Morula and Blastocyst Development Stages. Life.

[31] Zhao Z., Moley K.H., Gronowski A.M. (2013). Diagnostic Potential for MiRNAs as Biomarkers for Pregnancy-Specific Diseases. Clin. Biochem..

[32] Timofeeva A.V., Fedorov I.S., Sukhova Y.V., Ivanets T.Y., Sukhikh G.T. (2023). Prediction of Early- and Late-Onset Pre-Eclampsia in the Preclinical Stage via Placenta-Specific Extracellular MiRNA Profiling. Int. J. Mol. Sci..

[33] Timofeeva A.V., Fedorov I.S., Brzhozovskiy A.G., Bugrova A.E., Chagovets V.V., Volochaeva M.V., Starodubtseva N.L., Frankevich V.E., Nikolaev E.N., Shmakov R.G. (2021). MiRNAs and Their Gene Targets-A Clue to Differentiate Pregnancies with Small for Gestational Age Newborns, Intrauterine Growth Restriction, and Preeclampsia. Diagnostics.

[34] Pritchard C.C., Cheng H.H., Tewari M. (2012). MicroRNA Profiling: Approaches and Considerations. Nat. Rev. Genet..

[35] Gantier M.P., McCoy C.E., Rusinova I., Saulep D., Wang D., Xu D., Irving A.T., Behlke M.A., Hertzog P.J., Mackay F. (2011). Analysis of MicroRNA Turnover in Mammalian Cells Following Dicer1 Ablation. Nucleic Acids Res..

[36] McGoldrick E., Stewart F., Parker R., Dalziel S.R. (2020). Antenatal Corticosteroids for Accelerating Fetal Lung Maturation for Women at Risk of Preterm Birth. Cochrane Database Syst. Rev..

[37] Saccone G., Berghella V. (2016). Antenatal Corticosteroids for Maturity of Term or near Term Fetuses: Systematic Review and Meta-Analysis of Randomized Controlled Trials. BMJ.

[38] Pan J., Ho M. (2021). Role of Glypican-1 in Regulating Multiple Cellular Signaling Pathways. Am. J. Physiol. Cell Physiol..

[39] Panwar V., Singh A., Bhatt M., Tonk R.K., Azizov S., Raza A.S., Sengupta S., Kumar D., Garg M. (2023). Multifaceted Role of MTOR (Mammalian Target of Rapamycin) Signaling Pathway in Human Health and Disease. Signal Transduct. Target. Ther..

[40] Van Acker T., Tavernier J., Peelman F. (2019). The Small GTPase Arf6: An Overview of Its Mechanisms of Action and of Its Role in Host–Pathogen Interactions and Innate Immunity. Int. J. Mol. Sci..

[41] Shackelford D.B., Shaw R.J. (2009). The LKB1-AMPK Pathway: Metabolism and Growth Control in Tumour Suppression. Nat. Rev. Cancer.

[42] Kasprzak A. (2021). Insulin-Like Growth Factor 1 (IGF-1) Signaling in Glucose Metabolism in Colorectal Cancer. Int. J. Mol. Sci..

[43] Toussia-Cohen S., Castel E., Friedrich L., Mor N., Ohayon A., Levin G., Meyer R. (2024). Neonatal Outcomes in Pregnancies Complicated by Placenta Accreta- a Matched Cohort Study. Arch. Gynecol. Obstet..

[44] Perepelitsa S.A. (2020). Acute Respiratory Distress Syndrome in Preterm Newborns (Morphological Study). Gen. Reanimatol..

[45] Heidemann S.M., Nair A., Bulut Y., Sapru A. (2017). Pathophysiology and Management of Acute Respiratory Distress Syndrome in Children. Pediatr. Clin. N. Am..

[46] Johnson E.R., Matthay M.A. (2010). Acute Lung Injury: Epidemiology, Pathogenesis, and Treatment. J. Aerosol Med. Pulm. Drug Deliv..

[47] Ragaller M., Richter T. (2010). Acute Lung Injury and Acute Respiratory Distress Syndrome. J. Emerg. Trauma. Shock.

[48] Huang Q., Le Y., Li S., Bian Y. (2024). Signaling Pathways and Potential Therapeutic Targets in Acute Respiratory Distress Syndrome (ARDS). Respir. Res..

[49] Lu Q., Yu S., Meng X., Shi M., Huang S., Li J., Zhang J., Liang Y., Ji M., Zhao Y. (2022). MicroRNAs: Important Regulatory Molecules in Acute Lung Injury/Acute Respiratory Distress Syndrome. Int. J. Mol. Sci..

[50] Rajasekaran S., Pattarayan D., Rajaguru P., Sudhakar Gandhi P.S., Thimmulappa R.K. (2016). MicroRNA Regulation of Acute Lung Injury and Acute Respiratory Distress Syndrome. J. Cell. Physiol..

[51] Liu Y., Guan H., Zhang J.-L., Zheng Z., Wang H.-T., Tao K., Han S.-C., Su L.-L., Hu D. (2018). Acute Downregulation of MiR-199a Attenuates Sepsis-Induced Acute Lung Injury by Targeting SIRT1. Am. J. Physiol. Cell Physiol..

[52] Xu X., Liu X., Dong X., Yang Y., Liu L. (2022). MiR-199a-3p-Regulated Alveolar Macrophage-Derived Secretory Autophagosomes Exacerbate Lipopolysaccharide-Induced Acute Respiratory Distress Syndrome. Front. Cell. Infect. Microbiol..

[53] Deprez M., Zaragosi L.-E., Truchi M., Becavin C., Ruiz García S., Arguel M.-J., Plaisant M., Magnone V., Lebrigand K., Abelanet S. (2020). A Single-Cell Atlas of the Human Healthy Airways. Am. J. Respir. Crit. Care Med..

[54] Jardine L., Haniffa M. (2021). Human Lung Macrophages: Roll up for the MISTRG Tour. Immunity.

[55] Xu X., Liu X., Dong X., Qiu H., Yang Y., Liu L. (2022). Secretory Autophagosomes from Alveolar Macrophages Exacerbate Acute Respiratory Distress Syndrome by Releasing IL-1β. J. Inflamm. Res..

[56] Zhang S., Yin Y., Li C., Zhao Y., Wang Q., Zhang X. (2020). PAK4 Suppresses TNF-Induced Release of Endothelial Microparticles in HUVECs Cells. Aging.

[57] Mishra R., Benlhabib H., Guo W., Lerma Cervantes C.B., Mendelson C.R. (2018). Developmental Decline in the MicroRNA 199a (MiR-199a)/MiR-214 Cluster in Human Fetal Lung Promotes Type II Cell Differentiation by Upregulating Key Transcription Factors. Mol. Cell. Biol..

[58] Wright J.R. (2005). Immunoregulatory Functions of Surfactant Proteins. Nat. Rev. Immunol..

[59] Lawson P.R., Reid K.B. (2000). The Roles of Surfactant Proteins A and D in Innate Immunity. Immunol. Rev..

[60] Condon J.C., Jeyasuria P., Faust J.M., Mendelson C.R. (2004). Surfactant Protein Secreted by the Maturing Mouse Fetal Lung Acts as a Hormone That Signals the Initiation of Parturition. Proc. Natl. Acad. Sci. USA.

[61] Montalbano A.P., Hawgood S., Mendelson C.R. (2013). Mice Deficient in Surfactant Protein A (SP-A) and SP-D or in TLR2 Manifest Delayed Parturition and Decreased Expression of Inflammatory and Contractile Genes. Endocrinology.

[62] Gao L., Rabbitt E.H., Condon J.C., Renthal N.E., Johnston J.M., Mitsche M.A., Chambon P., Xu J., O’Malley B.W., Mendelson C.R. (2015). Steroid Receptor Coactivators 1 and 2 Mediate Fetal-to-Maternal Signaling That Initiates Parturition. J. Clin. Investig..

[63] Bird A.D., Flecknoe S.J., Tan K.H., Olsson P.F., Antony N., Mantamadiotis T., Mollard R., Hooper S.B., Cole T.J. (2011). CAMP Response Element Binding Protein Is Required for Differentiation of Respiratory Epithelium during Murine Development. PLoS ONE.

[64] Roos A.B., Berg T., Barton J.L., Didon L., Nord M. (2012). Airway Epithelial Cell Differentiation during Lung Organogenesis Requires C/EBPα and C/EBPβ. Dev. Dyn. Off. Publ. Am. Assoc. Anat..

[65] Garg M. (2015). Targeting MicroRNAs in Epithelial-to-Mesenchymal Transition-Induced Cancer Stem Cells: Therapeutic Approaches in Cancer. Expert Opin. Ther. Targets.

[66] Greening D.W., Gopal S.K., Mathias R.A., Liu L., Sheng J., Zhu H.-J., Simpson R.J. (2015). Emerging Roles of Exosomes during Epithelial-Mesenchymal Transition and Cancer Progression. Semin. Cell Dev. Biol..

[67] Bartels H.C., Postle J.D., Downey P., Brennan D.J. (2018). Placenta Accreta Spectrum: A Review of Pathology, Molecular Biology, and Biomarkers. Dis. Markers.

[68] Das V., Bhattacharya S., Chikkaputtaiah C., Hazra S., Pal M. (2019). The Basics of Epithelial–Mesenchymal Transition (EMT): A Study from a Structure, Dynamics, and Functional Perspective. J. Cell. Physiol..

[69] Kalluri R., Weinberg R.A. (2009). The Basics of Epithelial-Mesenchymal Transition. J. Clin. Investig..

[70] Chen C.-Y., Chen J., He L., Stiles B.L. (2018). PTEN: Tumor Suppressor and Metabolic Regulator. Front. Endocrinol..

[71] Makker A., Goel M.M., Nigam D., Mahdi A.A., Das V., Agarwal A., Pandey A., Gautam A. (2018). Aberrant Akt Activation During Implantation Window in Infertile Women With Intramural Uterine Fibroids. Reprod. Sci..

[72] Xiao J., Tao T., Yin Y., Zhao L., Yang L., Hu L. (2017). MiR-144 May Regulate the Proliferation, Migration and Invasion of Trophoblastic Cells through Targeting PTEN in Preeclampsia. Biomed. Pharmacother..

[73] Lou C.-X., Zhou X.-T., Tian Q.-C., Xie H.-Q., Zhang J.-Y. (2018). Low Expression of MicroRNA-21 Inhibits Trophoblast Cell Infiltration Through Targeting PTEN. Eur. Rev. Med. Pharmacol. Sci..

[74] Tian Y., Li H., Qiu T., Dai J., Zhang Y., Chen J., Cai H. (2019). Loss of PTEN Induces Lung Fibrosis via Alveolar Epithelial Cell Senescence Depending on NF-ΚB Activation. Aging Cell.

[75] Murrieta-Coxca J.M., Barth E., Fuentes-Zacarias P., Gutiérrez-Samudio R.N., Groten T., Gellhaus A., Köninger A., Marz M., Markert U.R., Morales-Prieto D.M. (2023). Identification of Altered MiRNAs and Their Targets in Placenta Accreta. Front. Endocrinol..

[76] Xue P., Zheng M., Diao Z., Shen L., Liu M., Gong P., Sun H., Hu Y. (2013). MiR-155* Mediates Suppressive Effect of PTEN 3’-Untranslated Region on AP-1/NF-ΚB Pathway in HTR-8/SVneo Cells. Placenta.

[77] Dini P., Daels P., Loux S.C., Esteller-Vico A., Carossino M., Scoggin K.E., Ball B.A. (2018). Kinetics of the Chromosome 14 MicroRNA Cluster Ortholog and Its Potential Role during Placental Development in the Pregnant Mare. BMC Genom..

[78] Dini P., El-Sheikh Ali H., Carossino M., Loux S.C., Esteller-Vico A., E. Scoggin K., Daels P., A. Ball B. (2019). Expression Profile of the Chromosome 14 MicroRNA Cluster (C14MC) Ortholog in Equine Maternal Circulation throughout Pregnancy and Its Potential Implications. Int. J. Mol. Sci..

[79] Xing Y., Fu J., Yang H., Yao L., Qiao L., Du Y., Xue X. (2015). MicroRNA Expression Profiles and Target Prediction in Neonatal Wistar Rat Lungs During the Development of Bronchopulmonary Dysplasia. Int. J. Mol. Med..

[80] Lv Y., Li Y., Wang J., Li M., Zhang W., Zhang H., Shen Y., Li C., Du Y., Jiang L. (2021). MiR-382-5p Suppresses M1 Macrophage Polarization and Inflammatory Response in Response to Bronchopulmonary Dysplasia through Targeting CDK8: Involving Inhibition of STAT1 Pathway. Genes Cells.

[81] Li Q., Zheng H., Chen B. (2023). Identification of Macrophage-Related Genes in Sepsis-Induced ARDS Using Bioinformatics and Machine Learning. Sci. Rep..

[82] Dang W., Tao Y., Xu X., Zhao H., Zou L., Li Y. (2022). The Role of Lung Macrophages in Acute Respiratory Distress Syndrome. Inflamm. Res..

[83] Locati M., Curtale G., Mantovani A. (2020). Diversity, Mechanisms, and Significance of Macrophage Plasticity. Annu. Rev. Pathol..

[84] Chen X., Tang J., Shuai W., Meng J., Feng J., Han Z. (2020). Macrophage Polarization and Its Role in the Pathogenesis of Acute Lung Injury/Acute Respiratory Distress Syndrome. Inflamm. Res..

[85] Shapouri-Moghaddam A., Mohammadian S., Vazini H., Taghadosi M., Esmaeili S.-A., Mardani F., Seifi B., Mohammadi A., Afshari J.T., Sahebkar A. (2018). Macrophage Plasticity, Polarization, and Function in Health and Disease. J. Cell. Physiol..

[86] Yang D., Wei H., Sheng Y., Peng T., Zhao Q., Xie L., Yang J. (2024). Circ_0006640 Transferred by Bone Marrow-Mesenchymal Stem Cell-Exosomes Suppresses Lipopolysaccharide-Induced Apoptotic, Inflammatory and Oxidative Injury in Spinal Cord Injury. J. Orthop. Surg. Res..

[87] Hellström A., Ley D., Hansen-Pupp I., Hallberg B., Löfqvist C., van Marter L., van Weissenbruch M., Ramenghi L.A., Beardsall K., Dunger D. (2016). Insulin-like Growth Factor 1 Has Multisystem Effects on Foetal and Preterm Infant Development. Acta Paediatr..

[88] Catalucci D., Latronico M.V.G., Condorelli G. (2008). MicroRNAs Control Gene Expression: Importance for Cardiac Development and Pathophysiology. Ann. N. Y. Acad. Sci..

[89] Neppl R.L., Wang D.-Z. (2014). The Myriad Essential Roles of MicroRNAs in Cardiovascular Homeostasis and Disease. Genes Dis..

[90] Joris V., Gomez E.L., Menchi L., Lobysheva I., Di Mauro V., Esfahani H., Condorelli G., Balligand J.-L., Catalucci D., Dessy C. (2018). MicroRNA-199a-3p and MicroRNA-199a-5p Take Part to a Redundant Network of Regulation of the NOS (NO Synthase)/NO Pathway in the Endothelium. Arterioscler. Thromb. Vasc. Biol..

[91] Lundberg J.O., Gladwin M.T., Weitzberg E. (2015). Strategies to Increase Nitric Oxide Signalling in Cardiovascular Disease. Nat. Rev. Drug Discov..

[92] The R Development Core Team A Language and Environment for Statistical Computing. R Foundation for Statistical Computing, Vienna, Austria. https://www.r-project.org.

[93] RStudio Team RStudio: Integrated Development for R. RStudio. http://www.rstudio.com/.

